# Hypoglycaemia in diabetes: do we think enough of the cause? An observational study on prevalence and causes of hypoglycaemia among patients with type 2 diabetes in an out-patient setting in Sri Lanka

**DOI:** 10.1186/s12902-018-0264-0

**Published:** 2018-06-08

**Authors:** H. A. Dissanayake, G. S. P. Keerthisena, K. K. K. Gamage, J. H. Liyanage, I. R. H. S. Ihalagama, W. M. U. A. Wijetunga, T. A. D. Tillekaratne, G. W. Katulanda, P. Katulanda

**Affiliations:** 1 Diabetes Research Unit, Department of Clinical Medicine, Faculty of Medicine, University of Colombo, Colombo, Sri Lanka; 2 Oxford Centre for Diabetes, Endocrinology and Metabolism, University of Oxford, Oxford, UK; 3 Cruddas Link Fellow, Harris Manchester University, University of Oxford, Oxford, UK; 4 Medical Research Institute, Colombo, Sri Lanka; 5 University Medical Unit, National Hospital of Sri Lanka, Colombo 10, Sri Lanka

**Keywords:** Hypoglycaemia, Diabetes, HbA_1C_, Native food

## Abstract

**Background:**

Hypoglycaemia is a feared experience for people with diabetes. We aimed to study the prevalence and causes of hypoglycaemia among Sri Lankans with diabetes.

**Methods:**

One thousand patients with diabetes attending a private sector diabetic clinic were interviewed using a structured questionnaire. Hypoglycaemic episodes within the preceding month were inquired and severity was graded according to clinical features and/or capillary blood glucose levels.

**Results:**

Mean age 55.0 years (± 12.5), 58.6% were males, mean diabetes duration 10.6 years (± 8.1), mean FPG 7.48 mmol/l (± 2.79) and mean HbA1c 7.82% (± 1.71) (62 mmol/mol). Of them, 26.1%. (mild 20.7%, moderate 3.9%, and severe 1.5%) experienced symptomatic hypoglycaemia. Sudden change diet (46.7%), unaccustomed exercise (15.7%) and increase in antihyperglycaemic therapy dosage (14.9%) were the recognized causes. Cause was not recognized by 16.3%. Non-prescribed native food items accounted for hypoglycaemia in 16.9% of patients (*Momordica charantia* 54.5%, *Costus speciosus* 52.3%, *Salacia prinoides* 11.4%, *Coccinia grandis* 6.8%, *Adenanthera pavonina* 4.5%). Severity of hypoglycaemia was positively correlated to age and duration of diabetes but not to HbA_1C_.

**Conclusion:**

Hypoglycaemia is common among patients with diabetes. Patients need advice on regular diet and exercise. Consumption of non-prescribed native foods should be considered as a possible cause.

**Electronic supplementary material:**

The online version of this article (10.1186/s12902-018-0264-0) contains supplementary material, which is available to authorized users.

## Background

Diabetes mellitus has reached epidemic levels in many countries, especially in Asia [1]. Disabling and life threatening micro and macrovascular complications of diabetes highlights the need for optimum management [2]. Benefits of intensive glycaemic control in prevention of these complications are well recognized [3, 4]. However it has been observed that most patients do not reach the optimum level of glycaemic control despite well defined management protocols [4]. This, at least partly, is attributable to the fear of hypoglycaemia, both among patients and treating medical professionals [5]. In fact, in the ACCORD study, the higher mortality rate in the intervention arm is largely attributable to higher incidence of hypoglycaemic events due to rapid reduction of blood glucose [6]. Unpleasant symptoms of hypoglycaemia reduce patients’ compliance to and confidence in treatment and discourage medical professionals in adopting aggressive treatment strategies [5]. Insulin and oral antihyperglycaemic agents, particularly those that increase insulin secretion independent of plasma glucose levels are the major causes for iatrogenic hypoglycaemia.

Incidence of hypoglycaemia has been reported up to 64%, varying with different settings and pharmacological therapies [7]. Furthermore, some studies have concluded that up to 50% of hypoglycaemic episodes are not recognized by patients [8] while only 15% of patients report recognized episodes to the doctor [8].

Studies on hypoglycaemia are limited and have yielded variable results due to difficulty in defining hypoglycaemia. It has been observed that recurrent hypoglycaemia lowers the plasma glucose threshold that produces symptoms [9–12] while persistently high plasma glucose levels raise it. This phenomenon probably explains the difficulty in defining a single cut-off for all patients hence the variations on blood glucose cut offs across different guidelines and research. Furthermore, such objective measures are not always available in managing patients on outpatient basis, particularly in resource poor settings. Nevertheless, an ADA and Endocrine Society workgroup concluded 70 mg/dl (3.9 mmol/l) of capillary blood glucose as an acceptable alert level for detection of hypoglycaemia [13]. This approximates the threshold for activation of counter regulatory mechanisms and sympathetic stimulation [13]. A capillary blood glucose level of 60 mg/dl (3.2 mmol/l) or less may produce neuroglycopaenic symptoms [5].

Although insulin and certain oral antihyperglycaemics are the most highlighted causes of hypoglycaemia, several other aetiologies and risk factors have been identified in various studies worldwide [5]. These include irregular eating habits like delaying meals or change in diet [14], alcohol use, unaccustomed physical activity, overdosing of insulin or oral antihyperglycaemics, critical illness [4, 5], and drug interactions [15]. Older age, longer duration of diabetes, concomitant renal disease, peripheral neuropathy, obesity, type 1 diabetes, advanced type 2 diabetes hypoglycaemic unawareness and cognitive dysfunction are the other risk factors recognized to predispose patients to hypoglycaemic episodes [7, 16–19].

Adverse effects of hypoglycaemia extend beyond acute symptoms of autonomic arousal and neuroglycopaenia [20–22]. In addition to causing death and irreversible brain damage [23] hypoglycaemia can also lead to long term consequences including weight gain (as a result of overeating to overcome symptomatic hypoglycaemic episodes) and subsequent deterioration of diabetes control, poor quality of life [5], increased cardiovascular morbidity and mortality [24, 25], electrocardiac dysfunction [4], cognitive impairment and dementia [26, 27]. Furthermore, a follow up study of ADVANCE trial showed an increased rate of microvascular and major macrovascular events, cardiovascular deaths and all cause mortality among patients with high incidence of hypoglycaemic episodes despite intensive glycaemic control [5].

Thus it is important to make patients aware of hypoglycaemia, its symptoms, common causes and measures to prevent such events. In fact, a study in the United Kingdom has shown significant benefit in providing health education to patient as well as family members on prevention, early recognition and appropriate management of hypoglycaemic episodes [28].

There is limited data from developing countries and we did not find any previous studies in Sri Lanka on prevalence of and causes for hypoglycaemia. Thus we aimed to study the prevalence of symptomatic hypoglycaemia among patients with diabetes mellitus and identify risk factors and causes.

## Methods

We studied a consecutive sample of 1000 patients with diabetes attending a private sector clinic from January 2013 to May 2013. People aged 18 year or older with diabetes diagnosed by a clinician based on American Diabetes Association criteria and commenced on treatment at least 3 month ago were selected. Pregnant and lactating women were excluded. A structured interviewer administered questionnaire was used by trained medical graduates maintaining uniformity (Additional file 1). Informed written consent was obtained from all participants. Patients were inquired about hypoglycaemic symptoms (dizziness, tremors, excessive hunger, sweating, clouding of consciousness) occurring within the previous month, documented glucometer reading of capillary blood glucose level or random blood glucose during such episodes and any recognized reason precipitating the hypoglycaemic episode. Information on glycaemic control (fasting venous blood glucose, HbA1c obtained from medical records), current pharmacological treatment, age, sex, and duration of diabetes were also recorded.

Occurrence of characteristics symptoms of hypoglycaemia that were corrected by glucose or refined carbohydrate consumption or dextrose administration was defined as a symptomatic hypoglycaemic episode. Severity was graded adopting American Diabetes Association criteria [29] (Table 1). Patient who was unresponsive to verbal commands and required parenteral administration of glucose were categorized as comatose. Glucometer reading of capillary blood glucose while being symptomatic was used for severity grading. If it was not available, clinical characteristics of the event were used. If capillary glucose level and clinical features were discrepant, patient was counted for the worst category. If a person had several hypoglycemic episodes of different severities, worst episode was counted for categorization. Most recent fasting venous plasma glucose and HbA_1C_ test results performed within last 3 month period were recorded.

**Table 1:** Definition of severity of hypoglycaemia

Severity of hypoglycaemia	Definition by clinical characteristics	Random capillary blood glucose value (mmol/l)
Mild	Symptoms of hypoglycaemia, patient recovers without external support	3.2–3.9
Moderate	Symptoms of hypoglycaemia, patient needs external support to recover	2.2–3.1
Severe	Comatose patient	< 2.2

Subsequently patients were directly inquired in to the cause of hypoglycaemia according to their perception. Any probable cause that occurred within a 24 h period prior to the hypoglycaemic episode was considered significant. These included change in meals (missing meals, unusual content / amount of a meal), unaccustomed exercise (any physical activity with exertion deemed out of proportion to ones usual daily activities), use of native food items, medication overuse, acute ill health or exacerbation of chronic diseases other than diabetes and overuse of medication were the common causes explored. Ethical approval was obtained from the Ethics Review Committee of Faculty of Medicine, University of Colombo.

### Statistical analysis

Data were tested for normality using visual inspection of the Q-Q plots and Shapiro-Wilk test. Kruskall Wallis test and Mann-Whitney U test were applied for non parametric data analysis while one-way ANOVA and student t test were used for parametric data. *P* values less than 0.05 were considered significant. Data analysis was performed using SPSS version 16 (SPSS Inc., Chicago, IL, USA).

## Results

Our survey population comprised of 1000 consecutive patients with diabetes mellitus seen at a private sector clinic. Mean age was 55.0 years (± 12.5), 58.6% were males, and mean duration of diabetes 10.6 years (± 8.0) (Table 2). Ninety eight percent of the participants had type 2 diabetes. Their mean fasting venous plasma glucose was 7.48 mmol/l (± 2.79), and mean HbA_1C_ was 62 mmol/mol (7.82%). Among these patients 29.7% were on insulin while the percentages of patients on metformin, sulfonylureas, pioglitazone, sitagliptin, GLP 1 agonist and acarbose were 83.9, 66.6, 2.8, 19.8, 0.9 and 2.2% respectively (most patients were on more than one antihyperglycaemic agent). Among these patients, 297 (29.7%) were on insulin while among those not on insulin, 193 (27.5%) were on a single oral antihyperglycaemic agent, 318 (45.3%) were two oral antihyperglycaemic agents and rest were on a combination of 3 or more.

**Table 2:** Demographic and clinical characteristics of the study population

Study population	1000
Age (years; mean ± SD)	55.0 (± 12.5)
Diabetes duration (years; mean ± SD)	10.6 (± 8.0)
Males	586 (58.6%)
Fasting plasma glucose (mmol/l; mean ± SD)	7.48 (± 2.79)
HbA1c (%, mean ± SD / mmol/l)	7.8 (± 1.7 / 62)

In this population 261 patients (26.1%) reported one or more symptomatic hypoglycaemic episodes within the preceding month. Among them, 15 (1.5%) had severe episodes and 39 (3.9%) had moderate episodes while the other 207 (20.7%) had only mild episodes. Capillary blood glucose data were available only in 53 (20.6, 11.2% mild, 5.4% moderate and 4.0% severe) of those who experienced symptomatic hypoglycemia.

Patients who had hypoglycaemic episodes of any severity had a higher mean age (*p* = 0.006) and a higher mean duration of diabetes (*p* < 0.001) compared to those without. Rising trends in age and duration of diabetes were observed with increasing severity of the episodes (Table 3) and the differences in mean age (*p* = 0.012) and diabetes duration (*p* < 0.001) were statistically significant. Pair wise Mann Whitney U test showed that the patients with severe hypoglycaemia were significantly older than those with no hypoglycaemia (*p* = 0.02). Those with mild (*p* < 0.001), moderate (*p* < 0.007) and severe (*p* = 0.001) hypoglycaemic episodes had significantly longer duration of diabetes than those had no hypoglycaemia.

**Table 3:** Age, male to female ratio, duration of diabetes, fasting venous plasma glucose and HbA_1C_ among patients without and with mild, moderate or severe hypoglycaemia (*N* = 1000)

	Patients without hypoglycaemia	Patients with hypoglycaemia				*P*_*1*_	*P*_*2*_
		Mild	Moderate	Severe	Total		
Number	739	207	39	15	261	–	–
Age (years)	55.0 (46.8–63.0)	57.0 (49.0–65.0)	59.0 (51.0–70.0)	63.0 (54.0–68.0)	57.0 (50.0–66.0)	0.006	0.012
M:F	1.4: 1	1.3: 1	1: 1	1.1: 1	1.2: 1		
Duration (years)	8.0 (4.0–15.0)	11.0 (5.0–17.3)	11.0 (6.0–17.0)	15.0 (10.0–22.0)	12.0 (6.0 -18.0)	< 0.001	< 0.001
FPG (mg/dL)	121.0 (103.0–150.1)	119.0 (98.0–141.3)	124.0 (96.0–148.0)	128.0 (81.0–162.0)	119.0 (97.0–143.0)	0.011	0.062
HbA1c (%) & (mmol/mol)	7.4 (6.5–8.8), 62	7.6 (6.8–8.6), 61	7.7 (7.1–9.1), 65	8.7 (7.7–9.5), 73	7.7 (6.7–8.7), 62	0.078	0.087

Age, duration, FPG and HbA1c expressed as median (25th and 75th percentiles)

*M:F* Male to female ratio, *FPG* Fasting plasma glucose, *HbA1c* Glycosylated haemoglobin A1c, *P*_*1*_ patients with no hypoglycaemia vs any form of hypoglycaemia (Kruskall Wallis test), *P*_*2*_: patients with no, mild, moderate or severe hypoglycaemia (Kruskall Wallis test)

Although the patients who had any form of hypoglycaemic episodes showed a slightly lower male to female ratio, this was statistically insignificant (*p* = 0.47) and no obvious pattern was observed with increasing severity of the episodes (Table 3).

Furthermore patients with hypoglycaemia of any severity had lower fasting plasma glucose levels compared to the others (*p* = 0.011). Although a falling trend in fasting plasma glucose was observed with increasing severity of the episodes, these differences were statistically insignificant (*p* = 0.062). Interestingly, in our population of patients, although statistically insignificant, those with hypoglycaemia showed a higher HbA_1C_ (*p* = 0.078) and a rising trend in HbA_1C_ was observed with increasing severity of hypoglycaemic symptoms (*p* = 0.087).

Out of the 261 patients who reported hypoglycaemic symptoms, 16.3% could not recognize a reason for such events. Sudden change in diet was the most common factor identified by patients as the cause for hypoglycaemic events (46.7%) (Table 4). This included delay in meals, skipping meals and significant change in the type of food. Unaccustomed physical activity was a cause recognized by 15.7% while excess hypoglycaemic therapy either due to recent dose increment or patient dependant factors (miscalculation of the dose, self treatment) accounted for 14.9%. Interestingly 16.9% of those who had hypoglycaemic episodes identified consumption of certain non-prescribed native food items as a hypoglycaemia precipitating factor. Furthermore, consumption of non-prescribed native food items was the only cause recognized in 10% of those who had hypoglycaemic episodes. Commonly used such food items include ‘Karawila’ (*Momordica charantia*) (54.5%), ‘Thebu’ (*Costus speciosus*) (52.3%), ‘Kothalahimbutu’ (*Salacia prinoides*) (11.4%), ‘Kowakka’ (*Coccinia grandis*) (6.8%) and ‘Madatiya’ leaves (*Adenanthera pavonina*) (4.5%). Although several patients had consumed more than one type of such food items, no significant association was observed between the number of food types and rate of hypoglycaemia or its severity.

**Table 4:** Recognized causes among patients with one or more hypoglycaemic episodes (*N* = 261)

Cause	Hypoglycaemia (%)			
	Mild	Moderate	Severe	Total
Sudden change in diet	45.9	51.3	46.7	46.7
Unaccustomed exercise	17.9	10.7	0.0	15.7
Native food	15.9	23.1	13.3	16.9
Excess medicine	13.0	23.1	20.0	14.9
Acute illness	2.4	2.6	0.0	2.2
Chronic illness	2.9	12.9	0.0	4.6
Other cause	2.0	0.0	0.0	1.6
No cause identified	17.2	13.2	13.3	16.3

Participants on sulfonylureas and/or insulin had higher rate of hypoglycaemia. For instance, 47.5% (58 out of 122) of those on both sulfonylurea and insulin had hypoglycaemic events while only 13.2% (21 out of 159) of those not on either had such events. Among those not on sulfonylurea and insulin, majority (89.2%) failed to identify a cause for hypoglycaemia while 3.2% attributed it to consumption of native food.

## Discussion

In this first large study on hypoglycaemia among Sri Lankan patients with diabetes we showed that one in four patients experienced symptomatic hypoglycaemic episodes within the preceding month. Patients who experienced hypoglycaemia had a lower fasting plasma glucose level and values showed a decreasing trend with increasing severity of the hypoglycaemic event. However, interestingly such patients had a higher HbA_1C_ and it showed an increasing trend with increasing severity of hypoglycaemic episodes. This might indicate that, in our population of patients, hypoglycaemia reduces treatment adherence. Or else, it may reflect the fact that those with poor glycaemic control were more likely to have their treatment up-titrated predisposing them to hypoglycemia. In fact this is comparable to observations in ACCORD study where attempts for stringent glycemic control was associated with more hypoglycaemic events [19]. Nevertheless this emphasizes that hypoglycaemia may still be prevalent among those with higher HbA_1C_ i.e., poor glycaemic control. However it is important to recognize that asymptomatic hypoglycaemic events may be more common, particularly among those with stringent glycaemic control, and these events might not have been recognized in our study. Furthermore, we analyzed only the severity of hypoglycaemia but not the frequency of hypoglycaemic events among patients with diabetes which may also be associated with strict glycaemic control and low HbA_1C_.

Large sample size is a strength of our study. Since a consecutive sample of patients managed on outpatient basis was studied it closely resembles a routine clinic setting. Our study also inquired into the effects of native food items, an aspect that has not been addressed in most studies. Most of these food items are vegetables, tubers and plant leaves that are used in Sri Lankan diet as well as in native medications. Their glucose content or mechanism of causing hypoglycemia is not known.

However this survey is limited by probable recall bias as it was dependant on patient reported hypoglycaemic episodes and recalling possible aetiology. Objective plasma glucose measurements were not available in most participants and available values were also derived from different glucometers that may have had variations in accuracy and precision. A continuous glucose monitoring system would have been ideal but is not feasible for an epidemiological study of this magnitude. Similarly, fasting venous plasma glucose and HbA1c tests were performed in different laboratories, on patients’ preference. Patients with asymptomatic hypoglycaemic episodes (for instance due to hypoglycaemic unawareness) may have gone undetected and therefore the true incidence of hypoglycaemia is likely to be higher than what we observed. Furthermore, impact of variations in native food item quantity, mode of preparation and frequency of consumption was not assessed in our survey. Relative risk of hypoglycaemia among participants who consume such native food items was not studied. Our study included only 20 people with type 1 diabetes and this did not allow a meaningful comparison of hypoglycaemia among people with type 1 and type 2 diabetes. Participants with at least one episode of hypoglycaemia were included for analysis irrespective of the frequency of events, which may also reflect the extent of hypoglycaemic risk. This aspect can be further explored in future studies.

Nevertheless, this survey highlights the need to consider the cause for hypoglycaemia in detail and importance of patient education in trying to identify the cause and avoid those in the future. It is important that patients adhere to a dietary pattern with minimum variations in energy content and to regular timing of meals. It is also important to adjust the diet and medicines to overcome the effect of unaccustomed exercise. All patients should be specifically inquired about hypoglycaemic events and helped to manage it successfully. Regular self monitoring of blood glucose is another simple but effective measure to recognize such events. For patients troubled with severe hypoglycaemic episodes, less stringent glycaemic targets as suggested in the latest ADA guidelines may be appropriate given the significant adverse outcomes [30]. Our study also highlights the need to conduct more research to explore the effects of non-prescribed native food items on plasma glucose levels with their potential benefit and harm on patients with diabetes.

## Conclusions and recommendations

In this first study on hypoglycaemia performed in Sri Lanka, we demonstrated that at least one in four patients with diabetes experience symptomatic hypoglycaemia during the preceding 1 month period. Severity of hypoglycaemic events was associated with advanced age, longer duration of diabetes and fasting blood glucose but not with HbA_1C_. Commonest cause for hypoglycaemia was sudden change in diet while unaccustomed exercise and non-prescribed native food were the other leading causes. These causes should be specifically looked into as a cause for hypoglycaemia particularly in the Sri Lankan and South Asian settings. Regular meal patterns and customizing drug regimens to variation in physical activity and concurrent illness should help to minimize the occurrence of this feared complication.

## Additional file

Additional file 1: Questionnaire - Identifying the causes of hypoglycaemia in people with diabetes. (DOCX 18 kb)

## Abbreviations

ADA: American Diabetes Association
HbA1c: Glycated haemoglobin A1c
