# Supplementary material for: Hypoglycaemia in diabetes: do we think enough of the cause? An observational study on prevalence and causes of hypoglycaemia among patients with type 2 diabetes in an out-patient setting in Sri Lanka
Source: BMC Endocr Disord. 2018 Jun 8;18:35. doi: 10.1186/s12902-018-0264-0 (PMC5994038; doi:10.1186/s12902-018-0264-0)
Supplement: Supplementary file 1 — Questionnaire - Identifying the causes of hypoglycaemia in people with diabetes. (DOCX 18 kb) [file 12902_2018_264_MOESM1_ESM.docx]

**Identifying the causes of hypoglycaemia in people with diabetes**

(Questionnaire to be administered by an interviewer)

1. **Demographic details**
   1. Age ………………………………………………………………………………..
   2. Sex Male / Female
   3. Year of diagnosis of diabetes ……………………………………………………….
   4. Type 1 2 other not known
   5. Most recent fasting blood glucose (within 1 month) ……………………………….
   6. Most recent HbA1c (within 3 months) ……………………………….
2. **Co-morbidities**
   1. Chronic kidney disease (eGFR < 60 mL/min/1.73m^2^ at least twice within last 3 months)
   2. Chronic liver disease (diagnosed by a physician based on clinical features, liver function tests and ultrasonography)
   3. Congestive cardiac failure (clinical syndrome with echocardiographic evidence of left ventricular ejection fraction 40% or less)
   4. Ischemic heart disease (documented evidence of acute coronary events)
   5. Other (specify) (COPD, Parkinsonism, dementia, malignancy etc) …………………………………………………………………………………… ………………………………………………………………………………………………………………………………………………………………………………………………………………………………………………………………………………………………………

**3. Hypoglycemic events**

**A. Within last 4 weeks;**

- 1. Did you experience any of these symptoms : sweating, dizziness, tremulousness,

excessive hunger, clouding of consciousness? Yes / No

- 1. Were they relieved by consumption of sugary food / drink / injection of

glucose? Yes / No

- 1. Did you require the assistance of another person to recover? Yes / No
  2. Did you lose consciousness during the event? Yes / No
  3. What was the random capillary blood glucose measured within 10 minutes of symptom

onset, before corrective measures were taken?

Value …………………….. mg/dL not available

(‘yes’ for 3.1 and 3.2 indicates confirmed hypoglycemic episode)

**B. Which of the following factors were temporally linked to above event(s)?**

(Occurred within the preceding 24h of the above event) (tick 1 or more)

- 1. Missed meal / delayed meal / unusually low quantity of meal
  2. Unaccustomed exercise
  3. Higher insulin dose than recommended / increment of insulin dose upon medical recommendation
  4. Higher dose / dose increment of a sulfonylurea
  5. Acute ill-health (febrile illness, hospitalization, acute illness necessitating a healthcare contact and specific treatment)
  6. Consumption of native food items:
     1. ‘Karawila’ (*Momordica charantia*)
     2. ‘Thebu’ (*Costus speciosus*)
     3. ‘Kothalahimbutu’ (*Salacia prinoides*)
     4. ‘Kowakka’ (*Coccinia grandis*)
     5. ‘Madatiya’ leaves (*Adenanthera pavonina*)
  7. Other (specify) ……………………………………………………………………………….. ………………………………………………………………………………………………………………………………………………………………………………………………
  8. None

**4. Current medication for diabetes** (indicate the dose consumed within last month)

4.1 Metformin

4.2 Sulfonylurea

4.2.1 Tolbutamide

4.2.2 Glibenclamibde

4.2.3 Gliclazide

4.2.4 Glipizide

4.2.5 Glimepiride

4.3 Pioglitazone

4.4 Acarbose

4.5 Insulin

4.5.1 Basal

4.5.2 Basal bolus

4.5.3 Premixed insulin twice daily

4.5.4 Premixed insulin thrice daily

4.6 GLP-1 agonists

4.6.1 Liraglutide

4.6.2 Exenatide

4.7 other (specify) …………………………………………………………………….. ………………………………………………………………………………………
